# Diabetes-Related Microvascular Complications in Primary Health Care Settings in the West Bank, Palestine

**DOI:** 10.3390/jcm12216719

**Published:** 2023-10-24

**Authors:** Mohammad Dweib, Nuha El Sharif

**Affiliations:** 1 College of Pharmacy and Medical Sciences, Hebron University, P.O. Box 40, Hebron P720, Palestine; mohammadd@hebron.edu; 2 School of Public Health, Al-Quds University, Abu Dis, P.O. Box 51000, Jerusalem 20002, Palestine

**Keywords:** diabetes, retinopathy, nephropathy, neuropathy, erectile dysfunction

## Abstract

Background: Worldwide, retinopathy, nephropathy, and neuropathy are the major diabetes-related microvascular complications. In Palestine, a low-middle-income country, diabetes is the fourth reason for death. However, a few studies examined diabetes microvascular consequences and its management. Therefore, we carried out a national study that aims to investigate the factors associated with diabetes-related microvascular complications among individuals seeking care in primary healthcare settings of the West Bank of Palestine. Method: Using a cluster systematic sampling technique, 882 participants with diabetes patients were chosen for a cross-sectional study from primary healthcare facilities operated by the Ministry of Health (PMoH), the United Nations Relief and Works Agency (UNRWA), and the Palestinian Medical Relief Society (PMRS). Data about patients related to diabetes-related complications, medication use, and other diseases were extracted from patients’ medical records. In addition, an interview face-to-face questionnaire was used to collect information about patients’ sociodemographic variables, medical history, smoking habits, duration of the disease, presence of concurrent conditions previous referrals, and hospital admissions, as well as their level of knowledge regarding diabetes, complications, and treatments. Results: Approximately 34.4% of persons with diabetes patients in Palestine encounter at least one microvascular complication associated with diabetes. The most prevalent diabetes-related microvascular complication was retinopathy (17.3%), 23.4% of participants had more than one microvascular complication, and 29% of male patients had erectile dysfunction. A higher probability of having any microvascular complications was associated with older age (over 60 years). Participants with diabetes patients with fundoscopy or ophthalmology reports, according to diabetes follow-up guidelines, were less likely to develop retinopathy. Also, those who performed regular kidney function testing were less likely to have nephropathy, and those who performed a regular foot exam were less likely to develop diabetic foot. Conclusions: Diabetes-related microvascular complications were associated with patient age, low education level, residency location, and adherence to diabetes follow-up guidelines of diabetes management; i.e., having been tested for HbA1c, consulting with specialists, regular kidney function, and foot examination. These factors can be utilized in setting up proper management protocols to prevent or delay microvascular complications in many patients.

## 1. Introduction

Poorly controlled chronic hyperglycemia is linked to both microvascular and macrovascular complications in individuals with diabetes [1]. Individuals diagnosed with diabetes are at an elevated risk of experiencing adverse health outcomes and mortality as a result of complications [2]. Microvascular complications tend to manifest at a faster rate and with greater frequency compared to macrovascular complications. Research suggests that individuals diagnosed with diabetes face a significantly elevated risk of microvascular disease, estimated to be 10–20 times greater compared to individuals without diabetes [3]. Research has also indicated that there is a notable disparity in the occurrence of microvascular problems among individuals diagnosed with Type 1 Diabetes Mellitus (T1DM) and Type 2 Diabetes Mellitus (T2DM) [4,5,6,7].

Individuals with T2DM exhibit a 2.24-fold higher susceptibility to diabetic retinopathy as compared to those with T1DM [8]. Retinopathy is observed in around 40–45% of individuals diagnosed with T1DM or T2DM [9]. Also, Koye et al. (2018) assert that diabetic peripheral nephropathy is the leading etiological factor contributing to the development of end-stage renal disease (ESRD) on a global scale [10]. Nephropathy is observed in around 40% of people diagnosed with either T1DM or T2DM [11]. Moreover, the prospect of lower limb amputation is regarded as one of the most dreaded consequences associated with diabetes. Furthermore, the presence of diabetic neuropathy might potentially give rise to the development of foot ulcers [12]. Moreover, it is worth noting that diabetic peripheral neuropathy tends to be more prevalent in those with T2DM [13,14,15,16,17,18] than in T1DM [19], and after a duration of 5 to 10 years of diabetes, the incidence of peripheral neuropathy approximately increases twofold [20,21]. Individuals diagnosed with diabetes are also prone to a higher likelihood of experiencing sexual difficulties. Kouidrat et al. (2017) established a strong association between diabetes and erectile dysfunction (ED) in men [13]. The prevalence of ED among males diagnosed with diabetes is approximately three and a half times greater compared to those in the general population [14,15,16,17,18,22].

Based on findings from the STEPWISE study, it was determined that the prevalence of diabetes among individuals aged 40 to 69 years within the Palestinian population in 2022 amounted to 20.8%. This prevalence was further disaggregated by gender, revealing rates of 17.3% among males and 24.3% among females. Additionally, data from the Palestinian PMOH indicated an incidence rate of 166.9 per 100,000 people for the year 2021 [23].

Several studies have investigated the microvascular complications of diabetes in the Palestinian population. The study conducted in Ramallah and Al Bireh governorate in 2012, titled “Palestinian Diabetes Complications and Control Study”, revealed that a total of 67% of individuals diagnosed with T2DM reported experiencing microvascular problems. The findings of the study indicated that the prevalence of these complications was positively associated with advancing age, higher levels of education, HbA1c levels exceeding 10, and residing in rural areas [24]. In another study, it was shown that 34.6% of individuals diagnosed with diabetes who were 35 years of age or older exhibited albuminuria. Specifically, 29.3% of these individuals displayed microalbuminuria, whereas 5.3% exhibited macroalbuminuria [25]. In a study conducted by Nazzal et al. (2020), it was demonstrated that a significant proportion of people with diabetes residing in the West Bank, specifically 23.6%, exhibited chronic renal illness. The likelihood of developing chronic renal illness was shown to be higher among older adults, smokers, and individuals with hypertension [26]. Our research involved conducting a nationwide survey in the West Bank region to investigate the factors associated with diabetes-related microvascular complications among individuals seeking care at primary healthcare facilities. The existing research has mostly concentrated on specific communities and used data from a single healthcare provider. As a result, we believe there is a need for more thorough, nationwide investigations that include every healthcare provider in order to draw generalized findings. As a result, the aim of this study is to fill these knowledge gaps by conducting a comprehensive analysis of diabetes-related microvascular complications, with a particular emphasis on the risk factors associated with diabetic retinopathy, peripheral neuropathy, diabetic nephropathy, and sexual dysfunction among individuals with T1DM and T2DM. Furthermore, we aimed to provide insights into the prevalence and contributing factors within the Palestinian population. This research seeks to inform policymakers about the necessary management strategies and protocols that, in turn, can improve the quality of life for people with diabetes.

## 2. Method

### 2.1. Study Context

The Palestinian Healthcare System encompasses various entities, including the government, non-governmental organizations (NGOs), UNRWA, Palestinian Military Medical Services (PMMS), and private healthcare services [27,28]. The responsibility for the provision of health care lies with the PMOH. The organization offers comprehensive healthcare services, encompassing primary, secondary, and tertiary levels of care, to the entirety of the population. However, it should be noted that the UNRWA exclusively provides primary healthcare services to refugees residing both within and outside the borders of Palestine [29]. The West Bank region encompasses a total of 669 primary health clinics and 51 hospitals, which include facilities operated by UNRWA as well as various humanitarian organizations [30].

To improve the quality of care of diabetes patients, the PMoH and the UNRWA, the two major stakeholders in Palestine, have developed guidelines for the management of diabetes; i.e., the UNRWA “Technical instructions and management protocols on the prevention and control of noncommunicable diseases”, and the PMoH Quick reference guide for the management and care of diabetes mellitus, which is called the “Quick Guide”. Both procedures adhere to the 2006 World Health Organization (WHO) diabetes care standards, although they may exhibit variations from each other and the aforementioned WHO guidelines [31].

### 2.2. Study Design and Population

The present research constitutes a cross-sectional study that examines the population of adult Palestinians residing in the West Bank who have been diagnosed with diabetes. In the Palestinian context, it is typical for individuals diagnosed with diabetes mellitus (DM) to be directed to the Primary Health Care (PHC) directorates located within their various regions. These PHC directorates serve as the primary providers of routine diabetic care, treatment, and monitoring for these patients.

Participating primary healthcare (PHC) facilities were selected using a stratified cluster random sampling method. Each of the three regions of the West Bank was represented by a governorate, namely, the northern, middle, and southern regions. In light of the involvement of the PMOH, UNRWA, and PMRS, a non-governmental organization, in the provision of basic healthcare services, our study employed a random selection process to identify one primary healthcare center (PHC) managed by each provider in every area. Consequently, our study incorporated a total of nine PHC institutions. A systematic sampling method was employed to choose participants from each primary healthcare center to be included in the study.

### 2.3. Sample Size

A calculated sample size of 900 patients was based on a confidence level of 95%, a predicted prevalence of diabetes-related microvascular complications among patients with diabetes of 20%, and a desired error margin of 5%. To accommodate the design effect (D = 2) resulting from the utilization of cluster samples by clinics’ location, the calculated sample size was divided equally between the three governorates. This sample of 300 patients was then distributed proportionally according to the size of the selected centers in each governorate. In each center, a random sample of patients’ records was selected and those patients were invited to participate on the day they visited the clinic.

### 2.4. Fieldwork

Individuals were classified as having diabetes if they had received a prior diagnosis of DM and were currently using injectable or oral hypoglycemic medications. The data were obtained through the patient’s records in a predetermined format. The data collection form encompassed several aspects of patients’ medical information, including their diagnoses, comorbidities, prescribed medications, monitoring tests, therapy received, and microvascular problems. In addition, a structured and pre-tested questionnaire was used to collect the data from patients through face-to-face interviews by trained interviewers. The questionnaire was based on questionnaires used previously in similar studies [32] with some modifications as suggested by the research team and by the major stakeholders themselves. The questionnaire comprised questions to assess patients’ background characteristics (age, gender, marital status, educational level, and address) and diabetes characteristics (family history of diabetes, type of diabetes patient medical regime, and ownership of a glucometer). Also, patients were asked about how their physicians cared for their diabetes in terms of laboratory tests (HbA1c, lipid profile, micro-albumin urea, kidney function testing); examinations (electro-cardiogram-ECG, foot examination, blood pressure measurements, and eye examination by ophthalmologist); and referral timing and the frequency of performing these tests and examinations. There were also questions on comorbid conditions: eye problems (retinopathy), extreme numbness (neuropathy), kidney problems (nephropathy), foot ulcer (diabetic foot), heart failure, hypoglycemia, and hypertension. Some questions related to the perception of patients on the follow-up of laboratory tests and examinations and diabetes comorbidities associated with their condition.

### 2.5. Diagnosis of Microvascular Complications

According to the clinical recommendations established by the Palestinian guidelines for diagnosing microvascular problems associated with diabetes, the primary method for identifying diabetic nephropathy includes performing kidney function tests and estimating the Glomerular Filtration Rate (GFR). These crucial tests are commonly conducted in both public and private healthcare institutions in order to guarantee precise diagnosis. To detect diabetic retinopathy, patients are referred to an ophthalmologist in a private medical practice or a private hospital. Patients suspected to have neuropathy are commonly referred to private neurologists for expert evaluation due to the intricate nature of the condition. Diabetic foot problems are typically identified with a clinical examination, which may be supplemented with Doppler investigations if deemed appropriate. In the event that Doppler testing is deemed necessary, patients are directed to a specialized private physician who possesses experience in the field of vascular assessment. Moreover, the identification of erectile dysfunction in individuals with diabetes predominantly relies on the subjective symptoms described by patients and the utilization of hormonal testing to ascertain the underlying factors.

### 2.6. Ethical Considerations

This study protocol was approved by the Helsinki Ethical Review Committee (code: PHRC/HC/20/15, approved on 3 June 2015). Written approval was obtained from the PMoH, UNRWA, and PMRS to access patients’ records. Prior to the interview, written informed consent was sought from participants after explaining the study objectives. Participants were reassured of the confidentiality of the collected data.

### 2.7. Data Analysis

Data were analyzed using Statistical Package for Social Sciences (SPSS)^®^ version 25. Categorical data were expressed as numbers (percentage) and continuous data as means ± standard deviation (SD) or median and range, as applicable. For data normality testing, histograms, Q-Q plots, and statistical tests such as the Shapiro–Wilk test, skewness, and kurtosis statistics were utilized. Bivariate analysis was performed using participants’ personal factors (i.e., socio-demographic and economic factors, family history of diabetes, and comorbidities) and diabetes management factors (i.e., healthcare provider, diabetes follow-up guidelines, and healthcare services factors) for each of the diabetes-related microvascular complications. A *p*-value of less than 0.05 is considered statistically significant. In addition, a multivariable logistic regression analysis was performed to determine the distinct association between the variables that were significant in the bivariate analysis and each of the diabetes-related microvascular complications, variables that are specific for the outcome microvascular complications from the follow-up testing, as well as other potential confounding factors. The adjusted odds ratios (AOR) and their 85% confidence intervals were computed.

## 3. Result

### 3.1. Characteristics of the Participants

As shown in Table 1, the study comprised a total of 882 participants, of whom 13.2% were diagnosed with T1DM, 71.4% with T2DM, and 15.4% did not have their diabetes type documented in their medical records. Furthermore, it is worth noting that approximately 36.4% of the individuals involved in the study were aged 60 or older. Additionally, half of the participants possessed an elementary level of education, while 57.4% of the sample identified as female. Of the participants, 72.1% reported a family history of diabetes, and 72% had type 2 diabetes; however, 15% had their diabetes type missed in their files. Surprisingly, 63.3% only utilized insulin. In addition, 55.8% of the participants had hypertension, 13.9% had dyslipidemia, 15.2% had coronary artery disease, and 22.1% were obese. 

### 3.2. Diabetes-Related Microvascular Complications and Other Comorbidities

**Figure 1:**
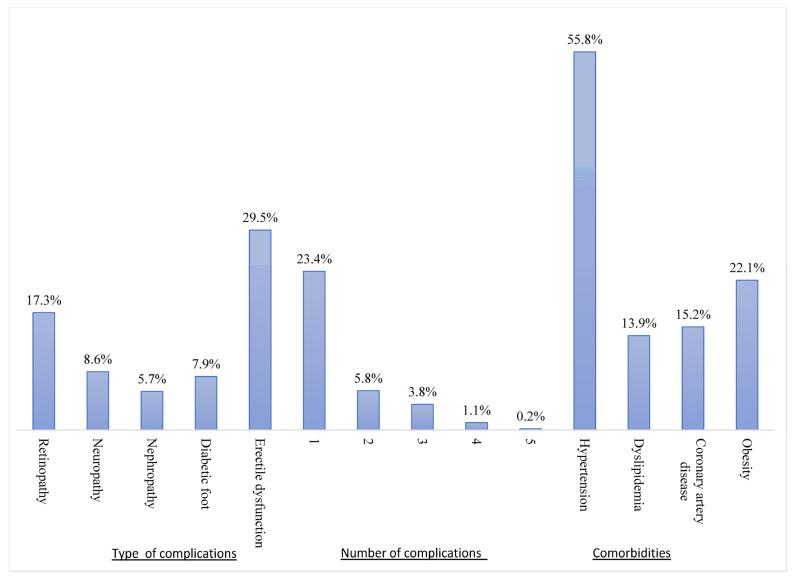
Distribution of diabetes-related microvascular complications and other comorbidities.

The present investigation revealed that 34.4% of the study participants exhibited the presence of at least one diabetic microvascular problem. Although erectile dysfunction (ED) was shown to be the prevailing microvascular problem among males with diabetes (29.5%), retinopathy was reported to be the most prevalent complication, accounting for approximately 17.3% among participants. Furthermore, it is worth noting that a significant proportion of the participants, specifically 85%, presented with at least one comorbidity (see Figure 1).

### 3.3. Management of Diabetes According to Guidelines

Based on the Palestinian follow-up guidelines for individuals with diabetes, a significant proportion of the patient records, specifically 75%, lacked the latest hemoglobin A1c (HbA1c) measurements, while 21% did not have the most recent fasting blood sugar (FBS) reading. Furthermore, it is noteworthy that a mere 17% of the patients’ files contained the latest ophthalmology report results, whereas a significantly higher proportion of 75% included the results of the renal function tests (Table 2).

### 3.4. Bivariate Analysis

A substantial association was seen between advanced age and the presence of retinopathy, neuropathy, nephropathy, and diabetic foot. Nevertheless, there was a substantial significant association between the educational attainment of patients and the presence of retinopathy, neuropathy, and diabetic foot (see Supplementary Table S1A,B).

Diabetes types, T1DM and T2DM, were shown to be significantly associated with rates of retinopathy, neuropathy, diabetic foot, and erectile dysfunction (*p* < 0.005). Also, the coexistence of hypertension as a comorbidity was found to have a substantial impact on the likelihood of developing neuropathy and nephropathy (*p* < 0.005). Similarly, the presence of dyslipidemia as a comorbidity was associated with neuropathy, nephropathy, and ED (*p* < 0.005). Also, having coronary artery disease was found to be significantly associated with all diabetes-related microvascular complications (*p* < 0.005). Interestingly, obesity was found to be significantly associated with developing diabetic foot (*p* < 0.005) (see Supplementary Table S2A,B).

Retinopathy was significantly associated with who diagnosed and followed the patient, the number of years of diagnosis, examined at each visit clinic, HbA1C testing as recommended, and history of hospitalization due to diabetes (*p* < 0.005). However, having nephropathy, neuropathy, and diabetic foot complications were associated with being tested for HbA1C as recommended (*p* < 0.05). Additionally, patients who have their BMI assessed during each visit demonstrate a significantly lower association with having diabetic foot complications (*p* < 0.05) (see Supplementary Table S3A,B).

### 3.5. Multivariate Analysis

The logistic regression model included disease characteristics, patient factors, and management-related factors as independent variables that were significantly associated with the microvascular complication in the Bivariate analysis. The model controlled for patient age, gender, place of residence, marital status, location of the primary healthcare center, and healthcare provider.

Table 3 shows that those aged 40 years and more had double the likelihood of obtaining retinopathy in comparison to their younger counterparts. Furthermore, individuals lacking basic literacy skills exhibited a higher likelihood of receiving a retinopathy diagnosis than those with a secondary education, diploma, or college education (AOR (education level > 12 years compared to illiterate: 0.45, 95% CI: 0.23–0.87). Moreover, it was shown that the inhabitants of the North governorate had a lower likelihood of having retinopathy compared to individuals residing in other governorates. Moreover, individuals who received specialized care had a considerably lower likelihood of developing retinopathy compared to those who received care from a general practitioner. Additionally, there is a notable difference in odds between individuals who have regular clinical examinations by their doctors during each visit and those who do not. Moreover, individuals who possess a fundoscopy or an ophthalmology report documented in their medical records (AOR: 2.004, 95% CI: 1.167–3.579), as well as those who adhere to recommended guidelines by regularly conducting these tests (AOR: 2.107, 95% CI: 1.34–3.314), show a notably higher likelihood of experiencing retinopathy compared to those lacking an ophthalmology report in their records. In addition, those who were registered at clinics operated by the PMoH exhibited a higher likelihood of experiencing retinopathy compared to those who sought medical care at clinics operated by UNRWA or other NGOs (see Table 3).

In Table 4, a high level of educational attainment exhibited a decreased likelihood of developing nephritis in comparison to patients lacking literacy skills. Additionally, the patients who had a documented HbA1c value over the past several months exhibited a decreased likelihood of developing nephropathy. Also, patients who adhere to proteinuria testing recommendations (AOR 2.574, 95% CI: 1.156–5.728), patients who maintain regular visits with an internist, and patients diagnosed with coronary artery disease (CAD) exhibit an increased likelihood of developing nephropathy. The study revealed notable disparities across healthcare providers and study regions.

In Table 5, the regression analysis showed that those patients in the age group 60 years or more exhibited a greater likelihood of experiencing neuropathy in comparison to those who were younger than 40 (AOR: 2.37, 95% CI: 1.159–4.846). Furthermore, the study revealed a strong association between the existence of a lipid profile in the patient’s medical records and an increased likelihood of neuropathy. Conversely, the presence of HbA1c results in the past 3 months was found to significantly decrease the probability of having neuropathy (AOR: 0.443, 95% CI: 0.233–0.845). In addition, regularly seeing a neurologist in accordance with guidelines was found to be associated with a higher likelihood of neuropathy diagnosis. Conversely, the presence of coronary artery disease (CAD) was associated with a decreased probability of experiencing neuropathy.

Table 6, the multivariate analysis, indicates a statistically significant increase in the likelihood of diabetic foot among patients aged 40 and above in comparison to those below the age of 40 years. Additionally, individuals who have been admitted to the hospital due to diabetes and those who have coronary artery disease (CAD) exhibit a higher likelihood of developing diabetic foot.

Table 7 presents the multivariate analysis of factors associated with erectile dysfunction. Results indicate that those who are not married exhibit a much greater likelihood of experiencing ED when compared to their married counterparts (AOR: 4.063, 95% CI: 1.147–14.39). Patients who engage in post-treatment consultations with a specialist exhibit a higher likelihood of experiencing ED in comparison to those who consult with a general practitioner. Also, the act of measuring blood pressure in accordance with guideline recommendations demonstrated a positive correlation with an increased likelihood of experiencing ED. Conversely, conducting an electrocardiogram (ECG) during the diagnostic process exhibited a negative correlation with the chance of experiencing ED. Individuals diagnosed with coronary artery disease (CAD) exhibited a decreased likelihood of experiencing ED.

## 4. Discussion

Based on the results of this study, it was determined that a total of 34.4% of the patients experienced the occurrence of at least one complication. The prevalence of retinopathy in individuals with diabetes was found to be 17%, making it the most frequently observed microvascular complication. Peripheral neuropathy, on the other hand, had a prevalence rate of 8.6%. However, it was found that a significant proportion of the male population, specifically 30%, experienced ED.

The escalating worldwide rates of mortality and morbidity associated with microvascular and macrovascular complications among individuals with DM are a matter of considerable apprehension. The significance of our work lies in the investigation of the incidence of diabetes-related microvascular problems and associated risk factors among the Palestinian community. The present study investigated the prevalence of microvascular complications among a sample of 882 outpatients, with 57% of the participants being female. The study was conducted in three distinct regions of the West Bank, Palestine. Based on the results of the study, it was determined that a total of 34.4% of the patients experienced at least one health issue. The most prevalent microvascular complication associated with diabetes was retinopathy, which affected 17% of individuals. This was followed by peripheral neuropathy, which affected 8.6% of individuals. Nevertheless, it was found that a significant proportion of the male population, specifically 30%, experienced ED.

### 4.1. Prevalence Rates of Diabetes-Related Complications

Several studies conducted in Palestine have reported varying prevalence rates of microvascular complications, ranging from 39% to 67% [26,33] which is much higher than our study findings. The study rates in our research exhibit a lesser magnitude in comparison to those observed in Jordan, although they demonstrate a greater level when compared to other economically developed Arabic nations such as Kuwait and Bahrain [34]. In a similar manner, our observed prevalence exhibited a lesser magnitude when compared to Australia, Colombia, and Iran although it displayed a larger magnitude in relation to the studies conducted in Ireland [35].

The prevalence of retinopathy was found to be 17.3% in the patient records; however, in the study conducted in the southern region of Palestine, it was reported to be 21.8% among the subjects [36]. The prevalence of retinopathy exhibited variation among different countries within the region, with Egypt reporting a rate of 41.5%, the Gulf countries observing a rate of 29.9%, and Tunisia documenting a rate of 8.1% [37,38]. According to a systematic review conducted on the incidence of diabetic retinopathy in the Eastern Mediterranean Region, the estimated prevalence of this condition was found to be 31% [35]. Multiple factors, such as population characteristics, sample size, and the diagnostic process employed, could potentially contribute to the observed variations.

The prevalence of nephropathy among the individuals was observed to be 5.7%. In contrast, the center region of Palestine exhibited a much higher prevalence rate of 51.4%, while the southern area demonstrated a lower prevalence rate of 4.9% [24,35]. Regionally, it was 40.8% in the United Arab Emirates [39], 42.5% in Oman [40] and 7% in Lebanon [41]. Between the years 2005 and 2008, the estimated prevalence of diabetic nephropathy in the United States was 34.5% [42]. The potential reasons for the variations in the incidence of kidney disease among patients from different countries could be attributed to the setting of our study, which took place in primary care centers, as opposed to other studies conducted in hospitals that primarily handle more severe cases referred to specialized departments such as endocrinology and nephrology.

The prevalence of neuropathy among the study participants was found to be 8.6%, which is lower than the stated frequency in the United Kingdom (21.7%) [43]. The prevalence of diabetic neuropathy in patients with T1DM in the Arab World was estimated to be 18% based on a meta-analysis [44]. The estimated prevalence of diabetic peripheral neuropathy among patients with T2DM in Jordan ranged from 36% to 39% [45,46]. The prevalence of diabetic foot was seen to be 7.9% among the participants in this particular study. In comparison, a separate study conducted in the southern region reported a prevalence rate of 7.5%, while the middle region exhibited a higher prevalence rate of 10%. The prevalence rate of diabetic foot in Egypt was reported to be 6.1% [47], whereas several districts in Saudi Arabia exhibited prevalence rates ranging from 1.8% to 19% [48,49,50]. In Jordan, the prevalence of diabetic foot ranged between 4% and 5.3% [51,52].

The prevalence of ED in this study was found to be lower (29.5%) compared to the reported rates in other nations. The prevalence of this condition was seen in 50% of males with diabetes in the United States, ranging from 35% to 78% in Mexico, 80% in Saudi Arabia, 41% in the Netherlands, 72.5% in Nigeria, 60% in India, and 77% in Iran [53,54,55,56,57]. In summary, it can be inferred that the incidence rates of neuropathy, diabetic peripheral neuropathy, diabetic foot, and ED in Palestine are relatively lower in comparison to numerous other nations. This observation implies that Palestine may have a comparably lesser burden associated with these particular health disorders.

### 4.2. Socio-Economic Factors and Microvascular Complications

This study revealed significant associations between age and participants’ educational level and several problems related to diabetes. The study identified several socio-economic characteristics that were found to be associated with an increased risk of developing DR [58,59,60]. The present study revealed a significant correlation between age and the likelihood of developing retinopathy, with those aged 40 years and older exhibiting a risk that is more than twice as high as their younger counterparts. This finding aligns with previous research inquiries that have also established a link between retinopathy and advancing age [61]. The likelihood of individuals developing DR seems to increase with advancing age [62], particularly in cases where individuals have been living with diabetes for an extended duration [63]. Prolonged hyperglycemia has been observed to induce retinal vascular impairment, resulting in the development of several stages of diabetic retinopathy [64]. Furthermore, our study revealed that individuals aged 60 years or older exhibited a probability of having a diabetic foot that was more than double that of younger patients. The potential cause of this phenomenon could be attributed to the presence of other comorbidities that elevate the susceptibility to diabetic foot, such as hypertension [65]. Alternatively, it may be attributed to age-related alterations in foot structure and function, as evidenced by previous research [66,67,68]. Furthermore, numerous studies have identified a significant correlation between education level and the occurrence of problems related to diabetes [69,70]. The findings of the present study have revealed a noteworthy and significant negative correlation between educational attainment and the prevalence of DR. Likewise, it was shown that educational attainment emerged as a significant predictor of nephropathy, implying that individuals with higher levels of education may have reduced susceptibility to nephropathy [71,72]. The observed phenomenon can be attributed to a heightened level of consciousness and comprehension of the imperative nature of diabetes management, as well as adherence to treatment protocols and lifestyle recommendations, as evidenced in the existing body of scholarly literature [73]. One plausible hypothesis posits that individuals with higher levels of education may possess enhanced opportunities for obtaining healthcare services, specifically in the realm of ocular health [74]. Moreover, those with a higher socioeconomic status may possess enhanced opportunities to obtain medical care [75] and are more likely to be capable of implementing lifestyle modifications that effectively control diabetes [76]. Additionally, it is worth noting that these patients may possess a heightened level of knowledge regarding the treatment and management of their condition [77]. This knowledge encompasses activities such as regular monitoring of blood glucose levels and adherence to recommended protocols [78]. Our study revealed a significant association between marital status and ED. The observed phenomenon can be attributed to emotional and psychological elements associated with marriage or having a spouse [79]. These characteristics are likely to foster a supportive and favorable atmosphere for sexual well-being [80].

### 4.3. Diabetes Monitoring, Follow-Up, and Diabetes-Related Microvascular Complications

The prevention and delay of diabetes and its associated consequences can be achieved through the provision of early identification and comprehensive care [81]. The results of our study indicate that diabetic patients who receive specialized care and undergo comprehensive clinical evaluations during each visit demonstrate a reduced likelihood of developing retinopathy in comparison to patients who are attended to by general practitioners or receive a less comprehensive assessment. Conversely, it was shown that patients who underwent treatment administered by specialists exhibited an increased likelihood of experiencing ED. The management of this particular instance typically requires a high level of knowledge, which is typically supplied by specialists rather than general practitioners [82,83]. This preference for specialist involvement may be attributed to either the complexity of the condition or the potential under-reporting of cases by patients who have been treated by general practitioners [84].

Patients who possess fundoscopy or ophthalmology records within their medical records exhibited a decreased probability of developing retinopathy. According to a study conducted in Saudi Arabia [85], it was suggested that maintaining regular follow-up appointments with an ophthalmologist is crucial in order to prevent the progression of DR and subsequent blindness. This strategy facilitates the implementation of timely interventions and the effective management of DR. In the Palestinian context, individuals with diabetes mostly receive medical care from general practitioners, which has the potential to impede the timely identification of DR. Recent research has demonstrated that the utilization of electronic medical record-based tools has been effective in identifying patients with diabetic retinopathy who have become disengaged from follow-up care. This presents healthcare practitioners with a valuable chance to promptly re-establish contact with these patients and ensure their continued involvement in the healthcare process [86].

The aforementioned findings underscore the significance of incorporating eye health evaluations into regular medical examinations, particularly for individuals who have an elevated likelihood of developing retinopathy [87]. Adherence to guideline recommendations for fundoscopy is of utmost significance as it guarantees that patients are provided with suitable screening and monitoring for retinopathy in accordance with published standards [88]. This practice plays a crucial role in mitigating or postponing the development and advancement of the condition [89]. The current literature has firmly established that many factors related to patient care, including adherence to frequent testing and the inclusion of test findings in the patient’s medical record, significantly contribute to reducing the likelihood of retinopathy [90].

Regular comprehensive ophthalmological examinations and fundoscopy can lead to early detection, proper intervention, and improved results of diabetic retinopathy [91]. In addition, the act of recording the findings from the ophthalmological assessment in the medical records of patients serves to strengthen the continuity of care [92]. This practice facilitates efficient communication among healthcare professionals [93] and adds to the improved management of diabetic retinopathy [94]. The adherence to evidence-based methods underscores the significance of proactive monitoring and comprehensive documentation in the optimization of retinopathy therapy, thereby mitigating the risk of blindness associated with this condition [95,96,97].

In addition, adhering to guideline recommendations by seeking consultation with an internist was found to be associated with a decrease in the occurrence of nephropathy. The potential cause of this phenomenon can be attributed to the specialized expertise [98] and proficiency of internists in managing diabetes and its associated complications [99,100].

In the present investigation, individuals who failed to comply with the diabetes treatment guideline recommendations pertaining to HbA1c testing exhibited a higher propensity for developing diabetic nephropathy.

According to recent studies conducted by Song et al. (2019) and Wang et al. (2019), adherence to guidelines for HbA1c testing has been found to have a significant impact on the development and progression of nephropathy. These findings suggest that regular monitoring and control of blood glucose levels can potentially mitigate the formation or advancement of nephropathy, which is commonly associated with hyperglycemia-induced glycation end products [101,102]. These end products, resulting from the glycation of proteins, collagen, lipids, and extracellular matrix components, have been implicated in the exacerbation of kidney dysfunction [103].

In contrast, it was observed that individuals with nephropathy had a higher level of adherence to guideline recommendations pertaining to the monitoring of proteinuria. According to McGrath and Edi (2019), the aforementioned statement suggests that the use of regular proteinuria screening and monitoring can contribute to the timely identification of nephropathy [104], hence facilitating prompt intervention and treatment [105].

Additionally. patients who performed HbA1c tests according to guideline recommendations were found to be less likely to have neuropathy, while patients who performed blood lipid tests according to guideline recommendations were more likely to have neuropathy. This adds to the importance of regular monitoring of such parameters to reduce the probability of neuropathy, as indicated in the literature [106,107].

The correlation between seeking medical consultation with a neurologist in accordance with guideline recommendations and the presence of neuropathy was observed to be significant. Neuropathy is a medical problem that necessitates specialized care and skill, which are exclusively possessed by professionals in the field of neurology [108,109].

Individuals who have been previously admitted to a hospital due to diabetes or its associated complications have a likelihood of developing diabetic foot that is more than twice as high. This phenomenon can be attributed to the presence of more severe diabetes and its associated consequences among these patients [110,111], or the possibility that they may have contracted an infection during their hospitalization. [112,113].

CAD had an association with all the complications studied. Having CAD was associated with a higher risk of retinopathy, diabetic foot, and nephropathy in our study. This may be explained by the underlying genetic predisposition [114] and risk factors such as hypertension and diabetes [115], or by overlapping pathophysiological pathways such as angiogenesis, neovascularization [116], and other factors including increased oxidative stress, platelet dysfunction, dyslipidemia and inflammation [117,118]. On the other hand, having CAD was associated with a lower probability of neuropathy and ED.

### 4.4. Limitations 

It is important to realize that the present study has certain limitations. The predictive power of the incidence of diabetes microvascular complications may be affected by the limited size of the study group. Furthermore, similar to other studies that rely on observation, there exists the possibility of under-coding of comorbidities, incomplete data, and errors in coding. Despite the implementation of many strategies to mitigate recall bias in this investigation, it remains plausible that this bias may have impacted the data obtained from the patients. Moreover, the inability to establish causation or temporal sequencing of variables is a limitation of this study, attributed to its cross-sectional methodology.

### 4.5. Implications

This study emphasizes the significance of accurately determining the incidence of diabetes complications within the Palestinian community. Such estimation can contribute to the improvement of patient monitoring and the reduction in disease exacerbation. The findings of the study indicate a prevalence rate of 34.4% for microvascular complications among patients with T2DM in Palestine. This prevalence rate is comparatively lower than some studies conducted in the same region but higher than others. These results emphasize the necessity for additional research endeavors and the establishment of standardized diagnostic tests in this field.

The findings of this study hold potential utility for healthcare practitioners in Palestine, as they can aid in the identification of individuals who are at an elevated risk of developing microvascular issues. The research additionally underscores the necessity for early identification and mitigation of these problems, given their substantial impact on both mortality and morbidity rates.

Furthermore, the results of this study have the potential to support healthcare providers in the formulation of efficacious management strategies and interventions aimed at addressing problems associated with diabetes. Healthcare professionals can utilize the results of this study to create screening programs and risk assessment tools that are tailored to identify individuals who would benefit from early intervention, including lifestyle modifications, medication modifications, and referrals to specialized care.

The findings of the study also suggest the necessity for improved management of comorbidities, particularly hypertension, among individuals with diabetes, given that hypertension represents a notable risk factor for the onset of microvascular problems. Therefore, it is imperative for healthcare providers to adopt a proactive approach in the management of hypertension among individuals with diabetes in order to mitigate the risk of additional problems.

In general, the findings of this study offer valuable insights into the incidence of diabetes complications and associated risk factors in Palestine. These insights can inform the formulation of efficient interventions aimed at preventing and managing microvascular complications in individuals with diabetes.

## 5. Conclusions

It is noteworthy that our research is the first in Palestine to provide insight into the prevalence of diabetes-related microvascular complications, as the sample size included patients from all geographical regions and the main primary healthcare providers.

The study emphasizes the crucial need for consolidation and implementation of better diabetes management services in Palestine. In addition, health professionals must give more importance to DM through early screening and health promotion. Older patients with long duration of DM and more comorbidities like hypertension and dyslipidemia must receive more advanced care. There is also a necessity for early detection and proper management of microvascular complications.

## Figures and Tables

**Table 1:** Characteristics of the participants.

Variable	Category	Frequency (Percentage)
Age	≤39	398 (45.1)
	40–59	163 (18.5)
	>60	321 (36.4)
	total	882
Gender	Male	376 (42.6)
	Female	506 (57.4)
	total	882
Marital status	Single	202 (22.8)
	Married	680 (77.2)
	total	882
Educational level	1–6 years	434 (49.2)
	7–12 years	327 (37.1)
	>12 years	121 (13.7)
	total	882
Place of living	Village	301 (34.2)
	City	469 (53.2)
	Refugee Camp	112 (12.6)
	total	882
Family history of diabetes	Positive	636 (72.1)
	Negative	231 (26.2)
	Do not know	15 (1.7)
	total	882
Diabetes type	Type 1	116 (13.2)
	Type 2	630 (71.4)
	Undefined	136 (15.4)
	total	882
Treatment type	Diet only	19 (2.2)
	Oral	380 (43.1)
	Oral and insulin	163 (18.5)
	Insulin	320 (63.3)
	total	882
Presence of hypertension	Yes	492 (55.8)
	No	390 (44.2)
	total	882
Presence of dyslipidemia	Yes	123 (13.9)
	No	759 (86.1)
	total	882
Presence of coronary artery disease (CAD)	Yes	134 (15.2)
	No	749 (84.8)
	total	882
Presence of obesity	Yes	195 (22.1)
	No	687 (77.9)
	total	882

**Table 2:** Distribution of availability of baseline and follow-up testing in patient files.

Information		Count	Percentage
Presence of FBS in the patient file for the last month	Yes	693	78.6
	No	189	21.4
	Total	882	
Presence of HbA1C test in the patient file for the last 3 months	Yes	226	25.6
	No	656	74.4
	Total	882	
Presence of lipid profile tests in the patient file for the last year	Yes	705	79.9
	No	177	20.1
	Total	882	
Presence of kidney function tests in the patient file for the last year	Yes	675	76.5
	No	207	23.5
	Total	882	
Presence of urine for microalbumin test in the patient file for the last year	Yes	194	22.0
	No	688	78
	Total	882	
Presence of a report from an ophthalmologist in the patient file for the last year	Yes	151	17.1
	No	731	82.9
	Total	882	
Presence of ECG in the patient file as a baseline	Yes	162	18.4
	No	720	81.6
	Total	882	

**Table 3:** Multivariate logistic regression for determinants of retinopathy.

	Retinopathy		Crude Analysis		Muti-Variate Analysis		
	Yes N (%)	No N (%)	COR	95% CI	Sig.	AOR	95% CI for AOR
Type of diabetes							
T1DM	12 (7.8)	104 (14.3)	REF.		0.035	REF.	
T2DM	94 (61.4)	536 (73.5)	1.52	0.80–2.87	0.773	0.894	0.416–1.919
Undefined	47 (30.7)	89 (12.2)	4.57	2.28–9.16	0.090	2.105	0.890–4.982
Age (years)							
≤39	42 (27.5)	356 (48.8)	REF.		0.003	REF.	
40–59	35 (22.9)	128 (17.6)	2.31	1.41–3.79	0.026	2.400	1.112–5.181
60	76 (49.7)	245 (33.6)	2.62	1.74–3.96	0.001	2.540	1.479–4.361
Educational level							
≤12 years	43 (28.1)	284 (39.0)	0.540	0.36–0.80	0.076	0.654	0.409–1.046
>12 years	15 (9.8)	106 (14.5)	0.505	0.28–0.90	0.018	0.450	0.232–0.873
Illiterate	95 (62.1)	339 (46.5)	REF.		0.033	REF.	
Who treats the patient?							
Specialist	286 (39.2)	44 (28.8)	0.62	0.42–0.91	0.000	0.316	0.166–0.603
GP	443 (60.8)	109 (71.2)	REF.			REF.	
The patient hospitalized because of DM							
Yes	63 (41.2)	183 (25.1)	2.09	1.45–3.00	0.012	1.720	1.124–2.632
No	90 (58.8)	546 (74.9)	REF.			REF.	
Physician examines patient clinically on each visit							
Yes	71 (46.4)	317 (43.5)	1.125	0.793–1.58	0.037	1.559	1.027–2.368
No	82 (53.6)	412 (56.5)				REF.	
Having phonoscopy or ophthalmology report							
Yes	24 (15.7)	284 (39.0)	3.430	2.16–5.43	0.012	2.044	1.167–3.579
No	129 (84.3)	445 (61.0)				REF.	
Frequency of fundoscopy							
According to guideline	91 (59.5)	268 (36.8)	2.52	1.76–3.60	0.001	2.107	1.340–3.314
Not according to the guideline	62 (40.5)	461 (63.2)	REF.			REF.	
Healthcare Provider							
MOH	73 (47.7)	346 (47.5)			0.003	REF.	
UNRWA	53 (34.6)	310 (42.5)	0.391	0.25–0.59	0.004	0.403	0.218–0.747
PMS	27 (17.6)	73 (10.0)	1.185	0.711–0.97	0.824	0.912	0.404–2.058
PHC location							
North governorate	20 (13.1)	280 (38.4)	REF.		0.000	REF.	
Middle governate	70 (45.8)	212 (29.1)	5.303	3.26–8.629	0.001	3.400	1.644–7.029
South governorate	63 (41.2)	237 (32.5)	1.769	1.03–3.017	0.000	4.281	2.099–8.731

COR crude odds ratio, AOR Adjusted odds ratio, REF.: Reference category, N: number of cases, %: perecnt of cases, CI: confidence interval. Variables in the logistic regression model: Patient age, patient gender, education level, place of living, primary healthcare provider, location of PHC, type of diabetes, medication type, family history of diabetes, having coronary artery disease, period of diagnosis, who treated the patient, who diagnosed the patient, presence of HbA1c test patient, presence of report from an ophthalmologist, presence of ECG in the patient file as a baseline, does the patient has his own glucometer, patient visits another PHC center for diabetes, patient visits a private clinic for diabetes, a patient hospitalized because of DM, familiarity with HbA1c, frequency of ordering HbA1c test, frequency of visiting your doctor for diabetes, frequency of blood sugar testing, frequency of HbA1c testing, frequency of kidney function testing, frequency of ECG testing, frequency of fundoscopy, frequency of internist visit, frequency of endocrinologist, taking antidiabetic drugs according to physicians prescription.

**Table 4:** Multivariate logistic regression for determinants of Nephropathy.

	Nephropathy		Crude Analysis		Muti-Variate Analysis		
	Yes N (%)	No (%)	COR	95% CI	Sig.	AOR	95% CI for AOR
Educational level							
Secondary	43 (28.1)	284 (39.1)	0.953	0.51–1.77	0.045	0.800	0.460–1.392
Diploma or college	15 (9.8)	106 (14.3)	1.005	0.42–2.38	0.430	0.213	0.062–0.728
Illiterate	95 (62.1)	339 (46.6)	REF.		0.014	REF.	
Presence of HbA1c test in the patient file for the last 3 months							
Yes	10 (20.0)	216 (26.0)	0.713	0.35–1.45	0.025	0.475	0.249–0.909
No	40 (80.0)	616 (74.0)	REF.		REF.		
Healthcare Provider							
MOH	26 (34.2)	393 (48.8)	REF.		0.001		
UNRWA	30 (39.5)	333 (41.3)	0.617	0.30–1.26	0.278	1.381	0.770–2.475
PMRS	20 (26.3)	80 (9.9)	3.437	1.73–6.82	0.000	4.107	1.933–8.723
PHC location							
North governorate	6 (7.9)	294 (36.5)	REF.		0.000	REF.	
Middle governate	21 (27.6)	261 (32.4)	0.000	5.188	0.000	6.748	2.525–18.032
South governorate	49 (64.5)	251 (31.1)	0.025	2.943	0.000	12.484	5.045–30.891
A patient has coronary artery disease							
Yes	34 (22.5)	100 (13.7)	3.82	2.09–6.99	0.000	3.060	1.700–5.500
No	119 (77.5)	629 (86.3)					
Frequency of proteinuria testing							
According to guideline	37 (74.0)	672 (80.8)	0.678	0.35–1.30	0.021	2.574	1.156–5.728
Not according to the guideline	13 (26.0)	160 (19.2)	REF.		REF.		

OR crude odds ratio, AOR Adjusted odds ratio, REF.: Reference category. Variables in the logistic regression model: Patient age, patient gender, marital status, education level, PHC location, family history of diabetes, clinic ownership, period of diagnosis, who treated the patient, who diagnosed the patient, medication type, diabetes type, presence of HbA1c test patient, presence of lipid test, presence of ECG in the patient file as a baseline, does the patient has his own glucometer, patient visits another PHC center for diabetes, patient visits a private clinic for diabetes, a patient hospitalized because of DM, home monitoring of blood sugar, familiarity with HbA1c, frequency of ordering HbA1c test, frequency of visiting your doctor for diabetes, physician exams patient clinically each visit, frequency of blood sugar testing, frequency of HbA1c testing, frequency of measuring blood pressure, frequency of lipid profile testing, frequency of kidney function testing, frequency of proteinuria testing, frequency of ECG testing, frequency of internist visit, frequency of endocrinologist, taking antidiabetic drugs according to physicians prescription, patient has HTN, patient has coronary artery disease, patient has obesity, patient has dyslipidemia.

**Table 5:** Multivariate logistic regression for determinants of Neuropathy.

	Neuropathy		Crude Analysis		Muti-Variate Analysis		
	Yes N (%)	No (%)	COR	95% CI	Sig.	AOR	95% CI for AOR
Age							
≤39	14 (18.4)	384 (47.6)	REF.		0.046	REF.	
40–59	24 (31.6)	139 (17.2)	4.730	2.38–9.41	0.291	1.637	0.655–4.090
60	38 (50.0)	283 (35.1)	3.680	1.96–6.92	0.018	2.370	1.159–4.846
Presence of HbA1c test in the patient file for the last 3 months							
Yes	16 (21.1)	210 (26.1)	0.757	0.42–1.34	0.013	0.443	0.233–0.845
No	60 (78.9)	596 (73.9)	REF.			REF.	
Presence of lipid profile test in the patient file for the last 3 months							
Yes	60 (78.9)	645 (80.0)	0.546	0.13–2.30	0.034	2.546	1.071–6.054
No	16 (21.1)	161 (20.0)					
Healthcare Provider							
MOH	21 (42.0)	398 (47.8)		REF.		REF.	
UNRWA	17 (34.0)	346 (41.6)	0.343	0.19–0.62	0.168	1.511	0.840–2.716
PMRS	12 (24.0)	88 (10.6)	0.219	0.11–0.42	0.000	5.046	2.369–10.750
PHC location (governorate)							
North	5 (10.0)	295 (35.5)	REF.		0.000	REF.	
Middle	19 (38.0)	263 (31.6)	5.188	2.11–12.7	0.001	5.729	2.132–15.393
South	26 (52.0)	274 (32.9)	2.943	1.14–7.57	0.000	10.89	4.072–29.131
Frequency of neurologist visits according to guideline							
Yes	2 (2.6)	6 (0.7)	3.604	0.72–18.1	0.026	8.327	1.295–53.564
No	74 (97.4)	800 (99.3)	REF.			REF.	
Having coronary artery disease							
Yes	25 (32.9)	109 (13.4)	0.319	0.19–0.53	0.001	0.373	0.208–0.668
No	51 (67.1)	697 (86.6)	REF.			REF.	

COR crude odds ratio, AOR Adjusted odds ratio, REF.: Reference category. Variables in the logistic regression model: Patient age, patient gender, marital status, education level, place of residence, type of diabetes, medication type, family history of diabetes, clinic ownership, period of diagnosis, who treated the patient, who diagnosed the patient, presence of HbA1c test patient, presence of ECG in the patient file as a baseline, does the patient has his own glucometer, patient visits another PHC center for diabetes, patient visits a private clinic for diabetes, home monitoring of blood sugar, familiarity with HbA1c, frequency of ordering HbA1c test, frequency of visiting your doctor for diabetes, frequency of neurologist visits according to the guideline, physician exams patient clinically each visit, frequency of blood sugar testing, frequency of HbA1c testing, frequency of measuring blood pressure, having coronary artery disease, frequency of ECG testing, frequency of internist visit, frequency of endocrinologist, taking antidiabetic drugs according to physicians prescription.

**Table 6:** Multivariate logistic regression for determinants of diabetic foot (DF).

	DF		Crude Analysis		Multivariate Analysis		
	Yes N (%)	No N (%)	COR	95% CI	Sig.	AOR	95% CI for AOR
Type of diabetes							
T1DM	2 (2.9)	114 (14.1)			0.012		
T2DM	51 (72.9)	579 (71.3)		1.20–20.91	0.219	2.659	0.558–12.660
Undefined	17 (24.3)	119 (14.7)	8.143	1.84–36.04	0.005	20.090	2.478–162.81
Age (years)							
≤39	11 (15.7)	387 (47.7)			0.013		
40–59	18 (25.7)	145 (17.9)	4.36	2.01–9.47	0.925	1.050	0.380–2.898
60	41 (58.6)	280 (34.5)	5.15	2.60–10.20	0.039	2.333	1.045–5.211
Patient hospitalized because of DM							
Yes	34 (48.6)	212 (26.1)	2.67	1.63–4.38	0.005	2.195	1.266–3.806
No	36 (51.4)	600 (73.9)					
Healthcare Provider							
MOH	28 (40.0)	391 (48.2)			0.019		
UNRWA	26 (37.1)	337 (41.5)	1.077	0.62–1.87	0.153	1.563	0.847–2.886
PMRS	16 (22.9)	84 (10.3)	2.660	1.37–5.13	0.005	3.032	1.397–6.583
PHC location (governorate)							
North	7 (10.0)	293 (36.1)			0.000		
Middle	19 (27.1)	263 (32.4)	3.024	1.25–7.30	0.732	0.754	0.150–3.787
South	44 (62.9)	256 (31.5)	7.194	3.18–16.25	0.000	6.811	2.721–17.045
Patient has coronary artery disease							
Yes	48 (68.6)	700 (86.2)	2.86	1.66–4.92	0.044	1.878	1.017–3.46
No	22 (31.4)	112 (13.8)					

COR crude odds ratio, AOR Adjusted odds ratio, REF.: Reference category. Variables in the logistic regression model: Patient age, patient gender, marital status, education level, place of residence, PHC location, clinic ownership, family history of diabetes, diabetes type, diabetes medication, period of diagnosis, who treated the patient, who diagnosed the patient, presence of HbA1c test patient, presence of lipid test, presence of ECG in the patient file as a baseline, does the patient has his own glucometer, patient visits another PHC center for diabetes, patient visits a private clinic for diabetes, a patient hospitalized because of DM, home monitoring of blood sugar, frequency of visiting your doctor for diabetes, physician exams patient clinically each visit, frequency of blood sugar testing, frequency of HbA1c testing, patient has coronary artery disease, patient has obesity, frequency of foot exam, frequency of internist visit, frequency of endocrinologist, taking antidiabetic drugs according to physicians prescription.

**Table 7:** Multivariate logistic regression for determinants of erectile dysfunction.

	Erectile Dysfunction		Crude Odds Ratio		Muti-Variate Analysis		
	Yes N (%)	No N (%)	COR	95% CI	Sig.	AOR	95% CI for AOR
Marital status of the patient							
Single	4 (5.2)	45 (15.1)	3.23	1.12–9.28	0.030	4.063	1.147–14.395
Married	73 (94.8)	254 (84.9)	REF.			REF.	
Who treat the patient							
Specialist	14 (18.2)	141 (47.2)	4.01	2.15–7.48	0.003	4.689	1.708–12.870
GP	63 (81.8)	158 (52.8)	REF.			REF.	
Frequency of measuring blood pressure							
According to guideline	63 (81.8)	230 (76.9)	0.741	0.39–1.40	0.035	2.825	1.075–7.422
Not according to guideline	14 (18.2)	69 (23.1)	REF.			REF.	
Frequency of ECG testing							
According to guideline	4 (5.2)	4 (1.3)	0.247	00.06–1.01	0.010	0.052	0.005–0.496
Not according to guideline	73 (94.8)	295 (98.7)	REF.			REF.	
Healthcare Provider							
MOH	33 (42.9)	168 (56.2)	REF.			REF.	
UNRWA	34 (44.2)	98 (32.8)	0.566	0.33–0.97	0.859	0.918	0.357–2.360
PMRS	10 (13.0)	33 (11.0)	0.648	0.29–1.44	0.002	0.113	0.028–0.463
PHC location (governorate)							
North	9 (11.7)	130 (43.5)	REF.			REF.	
Middle	61 (79.2)	54 (18.1)	0.061	0.028–0.13	0.000	0.025	0.009–0.073
South	7 (9.1)	115 (38.5)	1.137	0.41–3.15	0.232	2.078	0.627–6.890
Patient has coronary artery disease							
Yes	24 (31.2)	42 (14.0)	0.36	0.20–0.64	0.025	0.390	0.172–0.887
No	53 (68.8)	257 (86.0)	REF.			REF.	

COR crude odds ratio, AOR Adjusted odds ratio, REF.: Reference category. Variables in the logistic regression model: Patient age, marital status, education level, place of residence, family history of diabetes, clinic ownership, clinic location, period of diagnosis, who treated the patient, presence of HbA1c test patient, presence of lipid test, presence of ECG in the patient file as a baseline, does the patient has his own glucometer, patient visits another PHC center for diabetes, patient visits a private clinic for diabetes, home monitoring of blood sugar, frequency of visiting your doctor for diabetes, patient has coronary artery disease, physician exams patient clinically each visit, frequency of blood sugar testing, frequency of HbA1c testing, frequency measuring BMI, frequency of measuring blood pressure, frequency of ECG testing, frequency of foot exam, frequency of internist visit, frequency of endocrinologist, taking antidiabetic drugs according to physicians prescription.

## Data Availability

Data is unavailable due to privacy or ethical restrictions.

## Supplementary Materials

The following supporting information can be downloaded at: https://www.mdpi.com/article/10.3390/jcm12216719/s1; Table S1A: Distribution of microvascular complications (retinopathy, neuropathy, Nephropathy) by socio-demographic characteristicstitle; Table S1B: Distribution of microvascular complications (Erectile dysfunction, Diabetic foot) by socio-demographic characteristics; Table S2A: Distribution of microvascular complications (retinopathy, neuropathy, Nephropathy) by diabetes type, treatment type, family history and comorbidities; Table S2B: Distribution of microvascular complications (Erectile dysfunction, Diabetic foot) by diabetes type, treatment type, family history, and comorbidities; Table S3A: Distribution of microvascular complications (retinopathy, neuropathy, nephropathy) by management-related factors; Table S3B: distribution of microvascular complications (erectile dysfunction, Diabetic foot) by management-related factors.
