# Supplementary material for: Diabetes-Related Microvascular Complications in Primary Health Care Settings in the West Bank, Palestine
Source: J Clin Med. 2023 Oct 24;12(21):6719. doi: 10.3390/jcm12216719 (PMC10649955; doi:10.3390/jcm12216719)
Supplement: Supplementary file 1 [file jcm-12-06719-s001.zip › jcm-2663383-supplementary.pdf]

Table S1 A: Distribution of microvascular complications (retinopathy, neuropathy, Nephropathy) by socio-demographic characteristics

Variable	Category	Retinopathy			Neuropathy			Nephropathy		
		Yes	No	p-Value	Yes	No	p-Value	Yes	No	p-Value
**Age**	**<39**	42 (27.5)	356 (48.8)	0.00	14 (18.4)	384 (47.6)	0.00	11 (22)	387 (46.5)	0.006
	**40-59**	35 (22.9)	128 (17.6)		24 (31.6)	139 (17.2)		15 (30)	148 (17.8)	
	**>60**	76 (49.7)	245 (33.6)		38 (50)	283 (35.1)		24 (48)	297 (35.7)	
	**Total**	153	729		76	806		50	832	
**Gender**	**Male**	57 (37.3)	319 (43.7)	0.144	36 (47.4)	340 (42)	0.369	27 (54)	349 (41.9)	0.092
	**Female**	96 (62.7)	410 (56.3)		40 (52.6)	466 (58)		23 (46)	483 (58.1)	
	**Total**	153	729		76	806		50	832	
**Marital status**	**Single, widowed**	34 (22.2)	167 (22.9)	0.848	9 (11.8)	192 (23.9)	0.017	7 (14)	194 (23.3)	0.126
	**Married**	119 (77.8)	562 (77.1)		67 (88.2)	614 (76.1)		43 (86)	638 (76.7)	
	**Total**	153	729		76	806		50	832	
**Educational level**	**7-12**	43 (28.1)	284 (39.1)	0.002	25 (32.9)	302 (37.5)	0.01	18 (36)	309 (37.2)	0.984
	**>12 years**	15 (9.8)	106 (14.3)		3 (3.9)	118 (14.4)		7 (14)	114 (13.5)	
	**1-6**	95 (62.1)	339 (46.6)		48 (63.2)	386 (48.1)		25 (50)	409 (49.3)	
	**Total**	153	729		76	806		50	832	
**Place of living**	**Village**	67 (44.1)	234 (32.1)	0.001	30 (39.5)	271 (33.6)	0.54	18 (36)	283 (34.1)	0.961
	**City**	78 (50.7)	391 (53.7)		43 (56.6)	426 (52.9)		26 (52)	443 (53.3)	
	**Camp**	8 (5.3)	104 (14.1)		3 (3.9)	109 (13.4)		6 (12)	106 (12.7)	
	**Total**	153	729		76	806		50	832	
**PHC location**	Nablus	20 (13.1)	280 (38.4)	0.00	6 (7.9)	294 (36.5)	0.00	5 (10.0)	295 (35.5)	0.001
	Ramallah	70 (45.8)	212 (29.1)		21 (27.6)	261 (32.4)		19 (38.0)	263 (31.6)	
	Hebron	63 (41.2)	237 (32.5)		49 (64.5)	251 (31.1)		26 (52.0)	274 (32.9)	
	**Total**									
**Healthcare Provider**	**MOH**	73 (47.7)	346 (47.5)	0.015	26 (34.2)	393 (48.8)	0.00	21 (42.0)	398 (47.8)	0.014
	**UNRWA**	53 (34.6)	310 (42.5)		30 (39.5)	333 (41.3)		17 (34.0)	346 (41.6)	
	**PMRS**	27 (17.6)	73 (10.0)		20 (26.3)	80 (9.9)		12 (24.0)	88 (10.6)	
	**Total**									

Table S1 B: Distribution of microvascular complications (Erectile dysfunction, Diabetic foot) by socio-demographic characteristics

Variable	Category	Erectile dysfunction			Diabetic foot		
		Yes	No	p-Value	Yes	No	p- Value
**Age (years)**	**<39**	46 (59.7)	135 (45.2)	0.002	11 (15.7)	387 (47.7)	0.000
	**40-59**	3 (3.9)	60 (20.1)		18 (25.7)	145 (17.9)	
	**>60**	28 (36.4)	104 (34.8)		41 (58.6)	280 (34.5)	
	**Total**	77	299		70	812	
**Gender**	**Male**				35 (49.3)	341 (41.9)	0.236
	**Female**				35 (50.7)	471 (58.1)	
	**Total**				70	812	
**Marital status**	**Single, widowed**	4 (5.2)	45 (15.1)	0.022	12 (17.1)	189 (23.3)	0.237
	**Married**	73 (94.8)	254 (84.9)		58 (82.9)	623 (76.7)	
	**Total**	77	299		70	812	
**Educational level**	**Secondary, high school**	35 (45.5)	114 (38.1)	0.154	18 (25.7)	309 (38.1)	0.001
	**College, university**	22 (28.6)	72 (24.1)		3 (4.3)	118 (14.3)	
	**Illiterate or elementary**	20 (26.0)	113 (37.8)		49 (70)	385 (47.6)	
	**Total**	77	299		70	812	
**Place of living**	**Village**	31 (40.3)	88 (29.4)	0.036	30 (42.9)	271 (33.5)	0.025
	**City**	42 (54.5)	167 (55.9)		38 (54.3)	431 (53.0)	
	**Camp**	4 (5.2)	44 (14.7)		2 (2.9)	110 (13.5)	
	**Total**	77	299		70	812	
**PHC location**	Nablus	9 (11.7)	130 (43.5)	0.000	7 (10.0)	293 (36.1)	0.00
	Ramallah	61 (79.2)	54 (18.1)		19 (27.1)	263 (32.4)	
	Hebron	7 (9.1)	115 (38.5)		44 (62.9)	256 (31.5)	
	**Total**	77	299		70	812	
**Healthcare Provider**	**MOH**	33(42.9)	168(56.2)	0.106	28 (40.0)	391 (48.2)	0.006
	**UNRWA**	34(44.2)	98(32.8)		26 (37.1)	337 (41.5)	
	**PMRS**	10(13.0)	33(11.0)		16 (22.9)	84 (10.3)	
	**Total**	77	299				

Table S2 A: Distribution of microvascular complications (retinopathy, neuropathy, Nephropathy) by diabetes type, treatment type, family history and comorbidities.

Variable	Category	Retinopathy			Neuropathy			Nephropathy		
		Yes	No	p-Value	Yes	No	p-Value	Yes	No	p-Value
**Diabetes type**	T1DM	12 (7.8)	104 (14.3)	0.000	2 (2.6)	114 (14.2)	0.016	2 (4)	114 (13.7)	0.119
	T2DM	94 (61.4)	536 (73.5)		62 (81.6)	568 (70.4)		38 (76)	592 (71.2)	
	**Undefined**	47 (30.7)	89 (12.2)		12 (15.8)	124 (15.4)		10 (20)	126 (15.1)	
	**Total**	153	729		76	806		50	832	
**Treatment type**	**Diet only**	1 (0.7)	18 (2.5)	0.181	1 (1.3)	18 (2.2)	0.555	0 (0)	19 (2.3)	0.392
	**Tablet**	58 (37.9)	322 (44.1)		38 (50.0)	342 (42.3)		25 (50)	355 (42.6)	
	**Tablet and insulin**	34 (22.2)	129 (17.7)		14 (18.4)	149 (18.5)		6 (12)	157 (18.9)	
	**Insulin only**	60 (39.2)	260 (35.7)		23 (30.3)	297 (36.9)		19 (38)	301 (36.2)	
	**Total**	153	729		76	806		50	832	
**Family history of diabetes**	**Positive**	514 (70.5)	122 (79.7)	0.051	63 (82.9)	573 (71.1)	0.067	38 (76)	598 (71.8)	0.574
	**Negative**	203 (27.8)	28 (18.3)		13 (17.1)	218 (27.1)		12 (24)	219 (26.3)	
	**Don’t know**	12 (1.6)	3 (2.0)		0 (0.0)	15 (1.9)		0 (0)	15 (1.8)	
	**Total**	150	717		76	791		50	832	
**Hypertension**	**Yes**	92 (60.9)	400 (54.9)	0.172	51 (67.1)	441 (54.9)	0.041	37 (74)	455 (54.8)	0.008
	**No**	51 (39.1)	329 (45.1)		25 (32.9)	365 (45.1)		13 (26)	377 (45.2)	
	**Total**	153	729		76	806		50	832	
**Dyslipidemia**	**Yes**	28 (18.5)	95 (13)	0.077	19 (25)	104 (13)	0.004	19 (38)	104 (12.5)	0.000
	**No**	125 (81.5)	634 (87)		57 (75)	702 (87)		31 (62)	728 (87.5)	
	**Total**	153	729		76	806		50	832	
**CAD**	**Yes**	34 (22.5)	100 (13.7)	0.006	25 (32.9)	109 (13.4)	0.000	19 (38)	115 (13.9)	0.000
	**No**	119 (77.5)	629 (86.3)		51 (67.1)	697 (86.6)		31 (62)	717 (86.1)	
	**Total**	153	729		76	806		50	832	
**Obesity**	**Yes**	29 (19.2)	166 (22.8)	0.329	17 (22.4)	178 (22.1)	0.957	13 (26)	182 (22)	0.507
	**No**	124 (80.8)	563 (77.2)		59 (77.6)	624 (77.9)		37 (74)	650 (78)	
	**Total**	153	729		76	806		50	832	

Table S2 B: Distribution of microvascular complications (Erectile dysfunction, Diabetic foot) by diabetes type, treatment type, family history, and comorbidities.

Variable	Category	Erectile dysfunction			Diabetic foot		
		Yes	No	p- Value	Yes	No	p- Value
**Diabetes type**	**T1DM**	**8(10.4)**	**56(18.7)**	**0.000**	**2 (2.9)**	**114 (14.1)**	**0.007**
	**T2DM**	**42(54.5)**	**217(72.6)**		**51 (72.9)**	**579 71.3)**	
	**Undefined**	**27(35.1)**	**26(8.7)**		**17 (24.3)**	**119 (14.7)**	
	**Total**	**77**	**299**		**70**	**812**	
**Treatment type**	**Diet only**	1(1.3)	2(0.7)	0.174	0 (0)	19 (2.3)	0.581
	**Tablet**	36(46.8)	117 (39.1)		32 (45.7)	348 (42.7)	
	**Tablet and insulin**	8(10.4)	63 (21.1)		14 (20)	149 (18.4)	
	**Insulin only**	32(41.6)	117 (39.1)		24 (34.3)	296 (36.5)	
	**Total**	77	299		70	812	
**Family history of diabetes**	**Positive**	57(74.0)	204(68.2)	0.609	58 (82.9)	578 (71.1)	0.086
	**Negative**	19(24.7)	91(30.4)		12 (17.1)	219 (27.0)	
	**Don't know**	1(1.3)	4(1.3)		0 (0.0)	15 (1.8)	
	**Total**	77	299		70	797	
**Hypertension**	**Yes**	42(54.5)	154(51.5)	0.634	46 (66.7)	446 (54.9)	0.060
	**No**	35(45.5)	145(48.5)		24 (33.3)	366 (45.1)	
	**Total**	77	299		70	812	
**Dyslipidemia**	**Yes**	**20(26.0)**	**26(8.7)**	**0.000**	13 (18.8)	110 (13.6)	0.228
	**No**	**57(74.0)**	**273(91.3)**		57 (81.2)	702 (86.4)	
	**Total**	**77**	**299**		70	812	
**CAD**	**Yes**	**24(31.2)**	**42(14.0)**	**0.000**	**22 (31.9)**	**112 (13.8)**	**0.000**
	**No**	**53(68.8)**	**257(86.0)**		**48 (68.1)**	**700 (86.2)**	
	**Total**	**77**	**299**		**70**	**812**	
**Obesity**	**Yes**	**25(32.5)**	**32(10.7)**	**0.000**	10 (14.5)	185 (22.8)	0.112
	**No**	**52(67.5)**	**267(89.3)**		60 (85.5)	627 (77.2)	

	Total	77	299		70	812	

Table S3 A: Distribution of microvascular complications (retinopathy, neuropathy, nephropathy) by management-related factors.

		Retinopathy		p-Value	Neuropathy		p-Value	Nephropathy		p-Value
		Yes	No		Yes	No		Yes	No	
		N (%)	N (%)		N (%)	N (%)		N (%)	N (%)	
Who does the follow-up	GP	109(71.1)	443 (60.8)	0.018	53(69.7)	499(61.9)	0.175	36(72.0)	516(62.0)	0.157
	Specialist	44(28.9	286(39.2)		23(30.3)	307(38.1)		14(28.0)	316(38.0)	
	Total	153	729		76	806		50	832	
Who diagnosed the patient	GP	83(54.2)	388(53.4)	0.006	47(61.8)	424(52.7)	0.111	29(58.0)	442(53.1)	0.797
	specialist	46(30.1)	152(20.9)		18(23.7)	180(22.2)		10(20.0)	188(22.6)	
	Another	24 (15.7)	189 (25.9)		11(14.5)	202(25.1)		11(22.0)	202(24.3)	
	Total	153	729		76	806		50	832	
Years of diagnosis	<5	21(13.8	159(21.8	0.000	13(17.1)	167(20.7)	0.812	6(12.0)	174(20.9)	0.254
	5-9	43(28.3	238(32.7		26(34.2)	255(31.6)		14 (28.0)	267(32.1)	
	10-14	29(18.4)	172(23.5		16(21.1)	185(23.0)		13(26.0)	188(22.6)	
	>=15	60(39.5	160(22.0		21(27.6)	199(24.7)		17(34.0)	203(24.4)	
	Total	153	729		76	806		50	832	
Was the patient hospitalized	Yes	63 (41.2)	183 (25.1)	0.000	25(32.9)	221 (27.5)	0.312	25 (50.0)	221 (26.6)	0.000
	No	90 (58.8)	546 (74.9)		51 (67.1)	585 (72.5)		25 (50.0)	611 (73.4)	
	Total	153	729		76	806		50	832	
Physician exams patient clinically each visit	Yes	71 (47)	319 (43.5)	0.426	31 (40.8)	358 (44.3)	0.552	28 (56)	362 (43.4)	0.081
	No	80 (53)	412 (56.5)		45 (59.2)	448 (55.7)		22 (44)	470 (56.6)	
	Total	151	731		76	806		50	832	
having HbA1c (every 3	Does not remember	22 (13.8)	181 (24.3)	.012	7 (6.8)	196 (24)	.000	5 (6.3)	198 (23.4)	.016
	Yes	34 (22.4)	164 (22.7)		13 (17.6)	185 (23.1)		11 (22.9)	187 (22.6)	

months for T1DM and 6 months for T2DM)	**No**	**97 (63.8)**	**384 (53)**		**56 (75.7)**	**425 (52.9)**		**34 (70.8)**	**447 (54)**	
	**Total**	**154**	**729**		**77**	**805**		**50**	**832**	
BMI measurement every visit	Yes	122 (80.3)	549 (75.6)	0.220	57 (75)	614 (76.5)	0.764	32 (64)	639 (77.2)	.033
	No	31 (19.7)	180 (24.4)		19 (25)	192 (23.5)		18 (36)	193 (22.8)	
	Total	153	729		76	806		50	832	
Measuring blood pressure every visit	Yes	126 (82.9)	572 (78.8)	0.254	62 (82.7)	636 (79.2)	0.474	43 (86)	655 (79.1)	0.241
	No	27 (17.1)	157 (21.2)		14 (17.3)	170 (20.8)		7 (14)	177 (20.9)	
	Total	153	729		76	806		50	832	
Lipid profile testing every 3 months	Yes	7 (4.6)	54 (7.4)	0.209	8 (10.5)	53 (6.6)	0.196	2 (4)	59 (7.1)	.403
	No	146 (95.4)	675 (92.6)		68 (89.5)	753 (93.4)		48 (96)	773 (92.9)	
	Total	153	729		76	806		50	832	

Table S3 B: distribution of microvascular complications (erectile dysfunction, Diabetic foot) by management-related factors.

		Erectile dysfunction		p-Value	Diabetic foot		p-Value
		Yes	No		Yes	No	
		N (%)	N (%)		N (%)	N (%)	
**Who does the follow-up**	**GP**	**63(81.8)**	**158(52.8)**		46(65.7)	506(62.3)	
	**Specialist**	**14(18.2)**	**141(47.2)**	**0.000**	24(34.3)	306(37.7)	0.568
	**Total**	**77**	**299**		70	812	
Who diagnosed the patient	GP	38(49.4)	149(49.8)		38(54.3)	432(53.3)	
	specialist	13(16.9)	71(23.7)		19(27.1)	179(22.1)	
	Another	26(33.8)	79(26.4)	0.290	13(18.6)	200(24.7)	0.419
	Total	77	299		70	811	
Years of diagnosis	<5	14(18.2)	52(17.4)		10(14.3)	169(20.8)	
	5-9	17(22.1)	110(36.8)		25(35.7)	256(31.6)	
	10-14	21(27.3)	59(19.7)	0.090	18(25.7)	183(22.6)	0.572
	>=15	25(32.5)	78(26.1)		17(24.3)	203(25.0)	
	Total	77	299		70	811	
Was the patient hospitalized	Yes	25(32.5)	87(29.1)		**34(48.6)**	**212(26.1)**	
	No	52(67.5)	212(70.9)	0.564	**36(51.4)**	**600(73.9)**	**0.000**
	Total	77	299		**70**	**812**	
Physician exams patient clinically each visit	**Yes**	**27(35.1)**	**159(53.2)**		28 (40)	363 (44.5)	
	**No**	**50(64.9)**	**140(46.8)**	**0.031**	42 (60)	449 (55.5)	0.467
	**Total**	**77**	**299**		70	812	
Doctor asks the patient to perform HbA1c (every 3 months for T1DM and 6	Does not remember	10(13.0)	75(25.1)		**9 (10.3)**	**194 (23.4)**	
	Yes	19(24.7)	70(23.4)	0.069	**15 (22.1)**	**183 (22.7)**	**.030**
	No	48(62.3)	154(51.5)		**46 (67.6)**	**435 (53.9)**	
	Total	77	299		**70**	**812**	

months for T2DM)							
BMI measurement every visit	Yes	60(77.9)	221(73.9)	0.470	**46 (65.7)**	**625 (77.3)**	**.028**
	No	17(22.1)	78(26.1)		**24 (34.3)**	**187 (22.7)**	
	Total	77	299		**70**	**812**	
Measuring blood pressure every visit	Yes	63(81.8)	230(76.9)	0.356	52 (74.3)	646 (79.9)	0.262
	No	14(18.2)	69(23.1)		18 (25.7)	166 (20.1)	
	Total	77	299		70	812	
Lipid profile testing every 3 months	Yes	5(6.5)	26(8.7)	0.531	3 (4.3)	58 (7.2)	.365
	No	72(93.5)	273(91.3)		67 (95.7)	754 (92.8)	
	Total	77	299		70	812	
